# A Comparison of ddPCR and ARMS for detecting EGFR T790M status in ctDNA from advanced NSCLC patients with acquired EGFR‐TKI resistance

**DOI:** 10.1002/cam4.978

**Published:** 2016-12-20

**Authors:** Wenxian Wang, Zhengbo Song, Yiping Zhang

**Affiliations:** ^1^ Department of ChemotherapyZhejiang Cancer HospitalHangzhouChina; ^2^ Key Laboratory Diagnosis and Treatment Technology on Thoracic OncologyZhejiangChina

**Keywords:** ARMS, ddPCR, EGFR‐TKI, non–small cell lung cancer, T790M

## Abstract

A sensitive and convenient method for detecting epidermal growth factor receptor (EGFR) T790M mutations from circulating tumor DNA (ctDNA) in advanced non–small cell lung cancer (NSCLC) patients with acquired EGFR‐TKI resistance would be desirable to direct patient sequential treatment strategy. A comparison of two platforms for detecting EGFR mutations in plasma ctDNA was undertaken. Plasma samples and tumor samples were collected from patients with acquired EGFR‐TKI resistance in Zhejiang Cancer Hospital from December 2014 to December 2015. Extracted ctDNA was analyzed using two platforms (Droplet Digital PCR and ARMS [dPCR]). A total of 108 patients were enrolled in this study. One hundred and eight patient plasma samples were detected by ddPCR and 75 were detected by ARMS. And 16 patients obtained tissue re‐biopsy, using ARMS assay for detecting EGFR T790M mutation. In all, 43.7% (47/108) had acquired T790M mutation by ddPCR. In 75 patient plasma samples, comparing ddPCR with ARMS, the rates of T790M mutation were 46.7% (35/75) and 25.3% (19/75) by ddPCR and ARMS, respectively. Of all, 16 patients both had tumor and plasma samples, the T790M mutation rates were 56.3% (9/16) by ARMS in tissue and 50.5% (8/16) by ddPCR in plasma ctDNA. The progression mode tended to gradual progression in T790M mutation patients (40.4%), but the T790M negative was inclined to the mode of dramatic progression (39.3%). The patients with T790M‐positive tumors had a longer time to disease progression after treatment with EGFR‐TKIs (median, 13.1 months vs. 10.8 months; *P* = 0.010) and overall survival (median, 35.3 months vs. 30.3 months; *P* = 0.214) compared with those with T790M‐negative patients. Our study demonstrates ddPCR assay may provide a highly sensitive method to detect EGFR T790M gene in plasma. And T790M‐positive patients have better clinical outcomes to EGFR‐TKIs than T790M‐negative patients.

## Introduction

In recent years, epidermal growth factor receptor tyrosine kinase inhibitors (EGFR‐TKIs) are clinically effective in patients with non–small cell lung cancer (NSCLC) harboring sensitizing EGFR mutations. Eight randomized trials have demonstrated a significantly higher tumor response rate and longer progression‐free survival (PFS) in EGFR‐mutant patients treated with first‐line TKI 1, 2, 3, 4, 5, 6, 7, 8. However, a majority of patients eventually acquire resistance to the drug and experience disease progression 9, 10. The T790M mutation in the EGFR gene is regarded as the most common cause of acquired resistance to EGFR‐TKIs 11.

This T790M resistant mutation was found in approximately 50% of rebiopsy samples obtained from patients with acquired resistance to EGFRTKI therapy 11. However, this is challenging in clinical practice to obtain serial tumor rebiopsies following progression disease due to the invasiveness of the procedure, and also carries risk due to the limitations of the biopsy to reflect tumor heterogeneity and the evolution of genetic modifications 12, 13, 14, 15. Several studies showed tumor genome in plasma had homogeneity. 16, 17. Moreover, mutation detection in plasma has shown promise in terms of accessibility, convenience, and practicality compared with analysis of isolated circulating tumor cells 18, 19.

Detection rates of T790M ctDNA in plasma from NSCLC patients with acquired TKI resistance ranged from 30% to 50% using qualitative PCR‐based assays 20, 21, 22, 23.

Therefore, finding a sensitive detecting assay is important. We first used ddPCR assay and ARMS assay to evaluate the acquired T790M status in advanced NSCLC patients plasma. Nowadays, the association between acquired T790M status and the patients’ clinical outcome is still controversial. Furthermore, our study shows the clinical efficacy and sequential treatment strategy between EGFR T790M‐positive and ‐negative patients.

## Patients and Methods

### Patients

All patients with pathologically confirmed advanced or recurrent stage IV NSCLC with EGFR mutation, an Eastern Cooperative Oncology Group performance status of 0–2, acquired resistance to EGFR‐TKI (gefitinib, erlotinib, or icotinib) therapy. All patients received erlotinib, gefitinib, or icotinib orally at a recommended dose, either at first‐line therapy or after first‐line standard chemotherapy. Some patients received also second‐line chemotherapy before treatment with the TKI. Objective tumor responses were evaluated every 6–8 weeks in accordance with the Response Evaluation Criteria in Solid Tumors guidelines (version 1.1). And the acquired resistance to EGFR‐TKI was based on Jackman's clinical definition 24. The study was approved by the Ethic Committee of Zhejiang Cancer Hospital. Patients all have informed verbal consent to participate in the study.

### Plasma samples and tumor samples collection

Plasma samples (10 mL) were collected from all patients enrolled in our study, following progression on EGFR‐TKI treatment. We collected plasma samples when progression disease after EGFR‐TKI was observed according to RECIST 1.1 but a subsequent treatment did not begin. Plasma DNA was purified using a plasma cell–free DNA kit (AmoyDx, Xiamen, China). Plasma DNA was detected by Droplet Digital PCR (ddPCR) (AmoyDx, Xiamen, China) and amplification refractory mutation system (ARMS) (AmoyDx, Xiamen, China) assays, respectively. DNA was extracted from re‐biopsy tumor tissue (FFPE DNA kit, AmoyDx, Xiamen, China) and used ARMS assay to detect EGFR T790M mutation. All rebiopsy samples of DNA were extracted from tumor specimens as per standard protocols (FFPE DNA kit, AmoyDx, Xiamen, China) and were used for ARMS.

### TKI use after acquired resistance to initial EGFR‐TKI

After disease progression, we adopted the criteria defined by Yang et al. and divided the patients into three groups: local disease progression, gradual progression, and dramatic progression based on the duration of disease control, evaluation of tumor burden, and clinical symptoms 25. Local disease progression was defined as solitary progression with no more than three lesions, gradual progression was defined as minor increment of tumor burden, and dramatic progression was defined as rapid increment of tumor burden or with obvious cancer‐related symptoms.

### Statistical analysis

PFS1 was defined as the time from the start of EGFR‐TKI treatment to the first documentation of progressive disease (PD) or death from any cause, while PFS2 was defined as time from the date of the first PD by RECIST version 1.1 to the second PD or death. OS was defined as the period from diagnosed with advanced NSCLC to the date of death by any cause, or the date when the patient was last known to be alive. All time‐to‐event outcomes were estimated using the Kaplan–Meier method and compared across groups with the log‐rank test or the Cox proportional hazards model. Chi‐square and Fisher's exact tests were used to analyze correlations between EGFR status and statistical analysis was carried out with SPSS version 19.0 (SPSS, Inc., Chicago, IL). All statistical tests were two sided and results were considered significantly different if *P *<* *0.05.

### Follow‐up

All patients were followed up at the outpatient clinic or over phone and the last follow‐up time was in 26 February 2016

## Results

### Patients characteristics

From December 2014 to December 2015, 108 patients with activating EGFR mutation developed acquired resistance to EGFR‐TKI in our study. Seventy patients were with deletion in exon 19 and 33 patients had mutation in exon 21L858R, the other 5 patients harboring point mutation in 18G719X. Among them, 16 patients underwent rebiopsy. The median age was 57 years (range, 28–79 years). The percentage of males, never smokers, and patients with adenocarcinoma were 50.9%, 65.7%, and 94.4%, respectively (Table 1). For EGFR‐TKI treatment, 40.7% (44/108) patients received TKI as first‐line treatment and 59.3% (64/108) received second‐line or more. Of patients, 83 were treated with icotinib, 16 received gefitinb, and 5 with erlotinib. Among them, 38 patients had a local progression, 34 patients had gradual progression, and 36 patients had a dramatic progression. After acquired resistance to EGFR‐TKI, 38 patients received continuous TKI, 25 patients received continuous TKI plus chemotherapy, and 28 patients were switched to chemotherapy alone. However, there were 17 patients who did not get information in sequential treatment.

**Table 1:** Clinical characteristics and EGFR T790M ctDNA status in plasma of 108 patients

Features	ddPCR‐T790M (+)(*n *=* *47)	ddPCR‐T790M (−)(*n *=* *61)	*P*
Sex			0.115
Male	28 (50.9%)	27 (49.1%)	
Female	19 (35.8%)	34 (64.2%)	
Age			0.639
<65 years	11 (47.8%)	12 (52.2%)	
≥65 years	36 (42.4%)	49 (57.6%)	
Smoking			0.713
Yes	17 (45.9%)	20 (54.1%)	
No	30 (42.3%)	41 (57.7%)	
Stage			0.123
IIIB	0 (0%)	3 (100%)	
IV	47 (44.8%)	58 (55.2%)	
Pathology			0.172
Adenocarcinoma	46 (45.1%)	56 (54.9%)	
Nonadenocarcinoma	1 (16.7%)	5 (83.3%)	
Types of TKI			0.146
Erlotinib	6 (66.7%)	3 (33.3%)	
Gefitinib	9 (56.3%)	7 (43.8%)	
Icotinib	32 (38.6%)	51 (61.4%)	
Response to TKI			0.176
CR+PR	30 (49.2%)	31 (50.8%)	
SD	17 (36.2%)	30 (63.8%)	
PS			0.931
0–1	39 (43.3%)	51 (56.7%)	
2	8 (44.4%)	10 (55.6%)	
Types of EGFR mutation			0.709
19	31 (44.3%)	39 (55.7%)	
21L858R	14 (42.4%)	19 (57.6%)	
Others	2 (40.0%)	3 (60.0%)	
Progression modes			0.160
Dramatic progression	12 (33.3%)	24 (66.7%)	
Gradual progression	19 (55.9%)	15 (44.1%)	
Local progression	16 (42.1%)	22 (57.9%)	
Tumor size of progression			0.266
<4 cm	17 (51.5%)	16 (48.5%)	
≥4 cm	30 (40.0%)	45 (60.0%)	
Distant metastasis when resistance to TKI			0.935
Yes	12 (42.9%)	16 (57.1%)	
No	35 (43.8%)	45 (56.3%)	

EGFR, epidermal growth factor receptor.

### The results of EGFR T790M gene status

#### Comparing ddPCR assay with ARMS assay in the results of plasma T790M status

In our study, we respectively applied ddPCR and ARMS assays to detect EGFR T790M mutation status in 75 plasma samples. By ddPCR assay, the T790M mutation rate was 46.7% (35/75). However, by ARMS assay, the rate of T790M mutation was only 25.3% (19/75). ARMS assay detected T790M gene positive in 16 plasma samples, which were also positive by ddPCR assay (Table 2). Of the 19 patients, only 1 patient was below 5% abundance (1.44%) and all others were higher than 5% (5.90–27.76%). However, of the 16 patients, 11 patients were below 1% abundance (0.16–0.80%), 3 patients were below 2% but higher than 1% (1.56–1.81%), and 2 patients were higher than 5% abundance (5.09–7.19%).

**Table 2:** Comparing detection of T790M status by ddPCR assay with ARMS assay in 75 plasma ctDNA with resistance to EGFR‐TKI

ddPCR‐T790M status	ARMS‐T790M		Total
	Positive	Negative	
Positive	19	16	35 (46.7%)
Negative	0	40	40 (53.3%)
Total	19 (25.3%)	56 (74.7%)	75

#### Comparing ddPCR assay in plasma with ARMS assay in tissue T790M status

Here, we evaluated the detection sensitivity and specificity of plasma T790M mutation using the ddPCR assay in comparison with the T790M status in the paired tumor rebiopsy as standard. The T790M status in 16 pairs of tumor tissue and plasma from EGFR‐TKI relapsed NSCLC patients is summarized in Table 3. In ctDNA, the T790M mutation rate was 50.0% (8/16). In tumor tissue, the rate of T790M mutation was 56.3% (9/16). Among these patients, six were positive for T790M in both tumor tissue and plasma, and five were negative in both. Three patients were positive for T790M in tumor tissue, but negative in plasma. The other two patients were positive for T790M in plasma, but negative in tumor tissue. Compared with tumor tissue, the sensitivity and specificity for EGFR T790M mutation detection in plasma by ddPCR were 66.7% (6/9) and 71.4% (5/7), respectively.

**Table 3:** Comparing detection of T790M status by ddPCR from plasma ctDNA with ARMS from rebiopsy tissues in 16 patients with resistance to EGFR‐TKI

ddPCR‐T790M status	ARMS‐T790M in tissues		Total
	Positive	Negative	
Positive	6	2	8 (50.0%)
Negative	3	5	8 (50.0%)
Total	9	7	16

### The relationship between T790M mutation by ddPCR assay and clinical features

All 108 patients were detected by ddPCR from plasma ctDNA. In this study, 47 patients were positive for T790M and the frequency of T790M mutation was 43.5%. For 70 patients with EGFR 19 deletion, the mutation rate of T790M was 44.3% (31/70). And for 33 with 21L858R mutation, the rate was 42.4% (14/33). In the group of local progression, 16 patients had T790M‐positive (42.1%) and 22 patients had T790M‐negative (57.9%) mutations. However, in the group of gradual progression, the rate of mutation (55.9%) was compared to wild type (44.1%). On the contrary, in the group of dramatic progression, the frequency of negative (66.7%) mutation was higher than positive (33.3%).

### Association of ddPCR T790M mutation in acquired resistance to TKI plasma ctDNA with survivals on EGFR‐TKI

**Figure 1:**
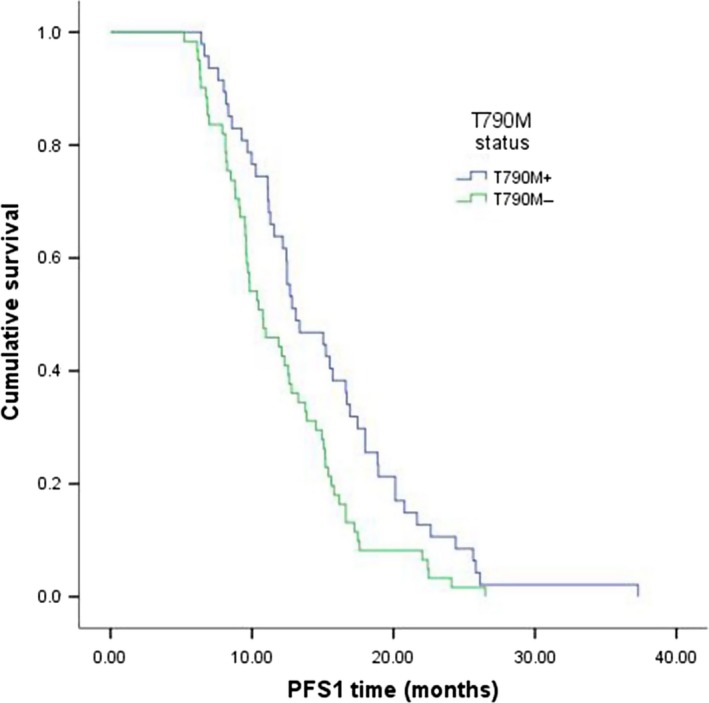
Kaplan–Meier curves showed that patients with T790M mutation had a significantly longer PFS1 than the group without T790M (13.1 months:10.8 months, *P* = 0.010).

PFS1 and OS were assessed in all 108 patients based on their plasma T790M status and TKI treatments. However, we did not access the subsequent treatment with 17 patients. In all patients, the median PFS1 was 12.3 months and the median OS was 32.8 months. For subgroup analysis, mPFS1 in the T790M‐positive group was longer than the negative group (13.1:10.8 months, *P* = 0.010) (Fig. 1). Similarly, the mOS in patients with T790M mutation was 35.3 months, which was significantly longer than 30.3 months in the patients without T790M mutation (*P *=* *0.214). On the other hand, we analyzed the clinical features between with T790M mutation and without T790M mutation in the time of PD. We found that the incidence rate of tumor size ≥4 cm in the patients with T790M‐negative mutation was slightly higher than patients with the T790M‐positive mutation (60.0%:40.0%, *P *=* *0.266). The occurrence rates of distant metastasis were similar (42.9%:43.8%, *P *=* *0.935). The difference in the median PFS1 (mPFS1) among the three groups was significant (*P *=* *0.006). The mPFS1 of patients in dramatic progression group (10.4 months) was shorter than that in gradual progression group (12.8 months) or local progression group (12.4 months).

**Figure 2:**
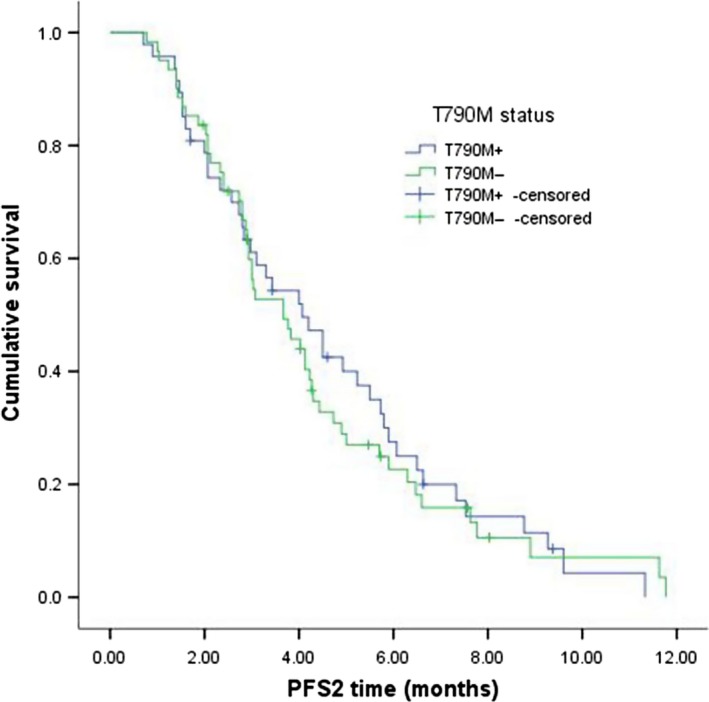
Kaplan–Meier curves showed that patients with T790M mutation had a slightly longer PFS2 than the subgroup without T790M mutation (4.0 months:3.1 months, *P* = 0.709).

Next, we investigated the sequential treatment of 91 patients after PD. The median PFS2 was 3.8 months (95% CI: 2.709–4.631). The PFS2 in the patients with T790M mutation was 4.0 months, which was not significantly longer than 3.1 months in the patients without T790M mutation (*P *=* *0.709) (Fig. 2). Among them, the PFS2 of 38 patients receiving continuous TKI was 3.0 months, PFS2 of 25 patients receiving continuous TKI plus chemotherapy was 5.7 months, and PFS2 of 28 patients who switched to chemotherapy alone was 2.9 months (Table 4). Subgroup analysis in group of receiving continuous TKI plus chemotherapy showed that PFS2 of T790M‐positive was longer than T790M‐negative patients (6.0: 4.3 months, *P *=* *0.722) (Table 5). Among the three groups of EGFR‐TKI failure modes, the PFS2 of patients in gradual progression group (6.0 months) was longer than that in dramatic progression group (4.9 months) or local progression group (4.3 months) (Table 6).

**Table 4:** The PFS2 of the sequential treatment in 91 patients after progression disease

Regimens	*n*	PFS (95% CI)	*P*
Continued TKI	38 (35.2%)	3.0 months (2.758–3.242)	
Chemotherapy	28 (25.9%)	2.9 months (2.579–3.361)	
Chemotherapy plus TKI	25 (23.1%)	5.7 months (3.053–8.407)	0.001

PFS, progression‐free survival.

**Table 5:** The relationship between acquired T790M status and sequential treatment with 91 patients

Therapeutic regimen	PFS2‐T790M (+)	PFS2‐T790M (−)	*P*
Continuous TKI	3.1 months	2.9 months	0.834
Chemotherapy	2.9 months	2.8 months	0.772
Continuous TKI plus chemotherapy	6.0 months	4.3 months	0.722

PFS, progression‐free survival.

**Table 6:** The relationship between progression mode and sequential treatment in 91 patients

Therapeutic regimen	Dramatic progression	Gradual progression	Local progression	*P*
Continuous TKI	3.3	2.1	3.1	0.066
Chemotherapy	3.0	3.0	2.1	0.512
Continuous TKI plus chemotherapy	4.9	6.0	4.3	0.440

## Discussion

Recently, with the study of overcoming acquired TKI resistance, it is becoming imperative to accurately and quickly detect molecular resistance mechanisms in individual patients. The secondary EGFR T790M mutation has been discovered in rebiopsy tumor tissue from about 50% of NSCLC patients with acquired resistance to EGFR‐TKI therapy 26, 27. Although detecting rebiopsy tumor tissue is a gold standard, the tissue may be difficult to obtain from resistance patients in some times. Therefore, detecting T790M status in patients’ plasma is an available method and a highly sensitive and convenient assay is needed, such as ARMS, ddPCR, and so on. Studies have showed that the sensitivity of ddPCR is 0.01% 28 and the sensitivity of the ARMS is 0.1% 29. Nowadays, a more standard and appropriate assay, to detect EGFR T790M status in plasma, is controversial. Therefore, we compared ddPCR with ARMS to clear that if ddPCR could provide an alternative approach in detecting plasma T790M status. On the other hand, the relationship between T790M mutation and patients’ prognosis is still controversial. Some researches demonstrated that T790M‐mutated patients were associated with poor survivals 30, 31, 32. However, other studies showed that NSCLC patients who acquired TKI resistance due to T790M gene mutation had longer survival than patients without T790M 33, 34. Therefore, we also analyzed ddPCR assay to detect plasma ctDNA T790M and T790M status associated with clinical outcome.

Nowadays, detecting EGFR gene status in plasma is a research focus. And some researchers have reported different assays on detecting EGFR in plasma 4, 21, 35, 36, 37, 38, 39. To further investigate the potential clinical role of detecting T790M gene statue in plasmas, we compared two major detecting assays which may be utilized frequently in clinical. The ARMS, which is based on quantitative PCR, identifies EGFR mutation sequences by using specific probes. With its high selectivity and sensitivity, this method can detect mutations in tissue samples containing as little as 1% mutated DNA 4. And it has been a standard method to detect EGFR gene status in tumor tissue. However, DNA in plasma was less than in tissue which needed more sensitive assay to detect EGFR gene status. Ma et al. 36 reported that EGFR mutation status tested by ARMS in plasma cannot replace a tumor tissue biopsy. The ARMS assay for EGFR mutation detection in plasma ctDNA has moderate sensitivity but high specificity. EGFR mutation positive results detected in plasma are fairly reliable, but negative results are hampered by a high rate of false negatives. ddPCR is a relatively new technology, requiring no external calibrators, for measuring the absolute and relative copy numbers of target DNA. Rapid microfluidic analysis of thousands of droplets per sample makes ddPCR practical for routine use 37. For real‐time PCR, the concentration of analyte was calculated by Ct comparison to the standard curve. ddPCR was direct quantitative analysis, but ARMS was indirect quantitative analysis. Thress et al. 38 compared four assays to detect EGFR T790M in ctDNA when patients had progression disease. The result showed that the sensitivity of ddPCR was higher than ARMS (71% vs. 29%). In our study, in the group of 75 plasma ctDNA, 19 were detected with T790M gene mutation by two assays and 16 were only detected with T790M mutation by ddPCR assay. And, in 19 patients, only 1 patient was below 5% abundance (1.44%). In the 16 patients, 11 patients were below 1% abundance (0.16%~0.80%), and 3 patients were below 2% but higher than 1% (1.56%~1.81%). Therefore, comparing ARMS assay, ddPCR is a potential method which had a higher precision, sensitivity, and accuracy to detect T790M gene status in plasma.

Takahama et al. [39] evaluated liquid biopsy assays for detection of TKI‐sensitizing and T790M mutations of EGFR by ddPCR in EGFR mutation positive NSCLC patients with acquired EGFR‐TKI resistance. And the result showed concordance for mutation detection by ddPCR in plasma relative to that in the paired samples with a sensitivity of 64.7% (20/31) and specificity of 70.0% (7/10) for T790M. Zheng et al. 33 studied the ddPCR method to dynamically detect EGFR mutations in patients’ plasma. The results demonstrated that T790M ctDNA mutation in plasma was detected in 55 (47%) of the 117 patients. And the overall concordance rate of T790M testing between the paired tumor tissues and plasma was 88.00% (22/25). The sensitivity and specificity of plasma T790M testing by ddPCR assay were 81.25% (13/16) and 100.00% (9/9), respectively. Lee et al. 40 used ddPCR to detect EGFR status in plasma from advanced NSCLC patients. The sensitivity and specificity of plasma EGFR testing by ddPCR assay were 74.1% and 100.00%, respectively. When compared with ARMS assay (25.3%) to detect the ctDNA acquired T790M, ddPCR had a higher sensitivity. Consistent with this, in our study of EGFR mutation NSCLC patient plasma samples, we detected T790M ctDNA by ddPCR in 43.7% (47/108) of patients with resistance to EGFR‐TKI therapy. And in our study, although only 16 patients experienced rebiopsy, the result showed that the sensitivity and specificity of plasma T790M testing by ddPCR assay were 66.7% (6/9) and 71.4% (5/7), respectively. Among them, six patients were with T790M‐positive mutation from tissues by ARMS assay, and two patients were without T790M mutation from plasma ctDNA by ddPCR method. We believe that biopsy is usually done at single site and it could not show the whole tumor pathological features and presence of the temporal and spatial heterogeneity of tumor tissue. The genetic information from patients’ plasma may be homogeneity in ctDNA. Therefore, detecting rebiopsy and blood samples may be mutual complementation in some cases. Furthermore, considering the feasibility and accessibility of blood sampling, we believe that the comprehensive profiling of TKI resistance to T790M mutation could be better performed using plasma ctDNA analysis of blood samples compared to single tumor rebiopsies. Hence, it needs more prospective studies to compare plasma with tissue in different situations.

Li et al. 32 demonstrated that patients with T790M mutation after acquired resistance to EGFR‐TKI benefit more from EGFR‐TKI treatment beyond progression compared to those without T790M mutation (13.0 vs. 10.5 months, *P* = 0.894) and had longer survival than T790M–negative patients (39.8 vs. 23.2 months, *P *=* *0.044). However, Zheng et al. 33 suggested patient's plasma T790M status upon TKI failure, which is associated with increased tumor burden and/or metastasis, thereby leading to poor survival (26.9 months vs. unreached, *P *=* *0.048). In our study, the PFS of T790M mutation patients was longer than patients without T790M (13.1 vs. 10.8 months, *P *=* *0.010) and the OS also was longer than T790M negative (35.3 vs. 30.3 months, *P *=* *0.214). Patients with T790M positive tended to local progression and gradual progression, but patients with T790M negative tended to dramatic progression. We also found that the incidence rate of tumor size ≥4 cm in the patients with T790M‐negative mutation was slightly higher than patients with the T790M‐positive mutation (60.0% vs. 40.0%, *P *=* *0.266). The tumor without T790M gene mutation may have a more tumor burden and rapid progression, or have other drive gene mutations, therefore would be poor survival.

Our study continued to analyze sequential treatment in patients with acquired resistance to EGFR‐TKI. The PFS2 in the patients with T790M mutation was slightly longer than patients without T790M mutation (4.0 vs. 3.1, *P *=* *0.709). And the PFS2 of patients receiving continuous TKI plus chemotherapy was better than other treatment agents. Among the three groups of EGFR‐TKI failure modes, the PFS2 of patients in gradual progression group (6.0 months) was longer than that in dramatic progression group (4.9 months) or local progression group (4.3 months). Trials including ASPIRATION 41 and IMPRESS 42 explored the treatment strategies for EGFR‐TKI failure. Continuing TKI plus chemotherapy might be a potential strategy beyond progression, and the algorithm will be answered by trial IMPRESS. IMPRESS showed that a subgroup analysis evaluating outcomes by T790M mutation status demonstrated a PFS advantage in T790M‐negative patients who received chemotherapy (cisplatin and pemetrexed) plus gefitinib versus only chemotherapy (6.7 vs. 5.4 months, *P* = 0.07), but not in T790M‐positive patients. However, in our study, 25 patients receiving chemotherapy plus continued TKI including single chemotherapy regimen was 21 (8 T790M positive and 13 T790M negative) and double chemotherapy regimen was 4. We thought that single chemotherapy not with platinum drugs might reverse TKI drug resistance, single chemotherapy plus TKI had an appropriate therapeutic strategy for T790M‐positive patients. Wei et al. 32 also analyzed the subsequent treatment after resistance to EGFR‐TKI, the results showed that the PFS2 in the patients with T790M mutation was 6.2 months, which was significantly longer than 2.6 months in the patients without T790M mutation (*P* = 0.002). Nowadays, several promising new agents such as CO1686 and AZD9291 that target T790M showed amazing results. And AZD9291 has been approved by FDA for the treatment of patients with metastatic EGFR T790M mutation‐positive NSCLC who have progressed on or after EGFR‐TKI therapy 43. However, in China, there is still no standard therapy for patients with T790M‐mediated TKI resistance in clinical practice. Thus, before the approval to use third TKI for T790M mutation, we could choose continuous TKI plus chemotherapy as first treatment agent.

We must mention that we have several limitations. First, because of the deficiency in majority of patient rebiopsy tissues, we had not compared all ctDNA with tumor tissues. Tumor tissues in 16 samples were analyzed by immunohistochemistry (IHC), detected T790M gene status by ARMS, and residual tissues were deficient in detection by ddPCR compared with the plasmas. Second, the nature of retrospective study will induce the statistical bias. And the physicians’ decision regarding continuation of TKI or switching to chemotherapy might influence result of observational analysis.

In conclusion, this study indicated the advantages of ddPCR in the associated EGFR T790M mutation after resistance to EGFR‐TKI. It is expected that ddPCR may have good application prospects and may play an increasing role in patients with acquired EGFR T790M mutation along with the research development of third EGFR‐TKI.

## Conflict of Interest

None declared.

## References

[1] Rosell, R. , E. Carcereny , R. Gervais , A. Vergnenegre , B. Massuti , E. Felip , et al. 2012 Erlotinib versus standard chemotherapy as first‐line treatment for European patients with advanced EGFR mutation‐positive non‐small‐cell lung cancer (EURTAC): a multicentre, open‐label, randomised phase 3 trial. Lancet Oncol. 13:239–246. 22285168 10.1016/S1470-2045(11)70393-X

[2] Zhou, C. , Y. L. Wu , G. Chen , J. Feng , X. Q. Liu , C. Wang et al. 2011 Erlotinib versus chemotherapy as first‐line treatment for patients with advanced EGFR mutation‐positive non‐small‐cell lung cancer (OPTIMAL, CTONG‐0802): a multicentre, open‐label, randomised, phase 3 study. Lancet Oncol. 12:735–742. 21783417 10.1016/S1470-2045(11)70184-X

[3] Mitsudomi, T. , S. Morita , Y. Yatabe , S. Negoro , I. Okamoto , J. Tsurutani , et al. 2010 Gefitinib versus cisplatin plus docetaxel in patients with non‐small‐cell lung cancer harbouring mutations of the epidermal growth factor receptor (WJTOG3405): an open label, randomised phase 3 trial. Lancet Oncol. 11:121–128. 20022809 10.1016/S1470-2045(09)70364-X

[4] Fukuoka, M. , Y. L. Wu , S. Thongprasert , P. Sunpaweravong , S. S. Leong , V. Sriuranpong , et al. 2011 Biomarker analyses and final overall survival results from a phase III, randomized, open‐label, first‐line study of gefitinib versus carboplatin/paclitaxel in clinically selected patients with advanced non‐small‐cell lung cancer in Asia (IPASS). J. Clin. Oncol. 29:2866–2874. 21670455 10.1200/JCO.2010.33.4235

[5] Maemondo, M. , A. Inoue , K. Kobayashi , S. Sugawara , S. Oizumi , H. Isobe , et al. 2010 Gefitinib or chemotherapy for non‐small‐cell lung cancer with mutated EGFR. N. Engl. J. Med. 362:2380–2388. 20573926 10.1056/NEJMoa0909530

[6] Miller, V. A. , V. Hirsh , J. Cadranel , Y. M. Chen , K. Park , S. W. Kim , et al. 2012 Afatinib versus placebo for patients with advanced, metastatic non‐small‐cell lung cancer after failure of erlotinib, gefitinib, or both, and one or two lines of chemotherapy (LUX‐Lung 1): a phase 2b/3 randomised trial. Lancet Oncol. 13:528–538. 22452896 10.1016/S1470-2045(12)70087-6

[7] Sequist, L. V. , J. C. Yang , N. K. Yamamoto , K. O'Byrne , V. Hirsh , T. Mok , et al. 2013 Phase III study of afatinib or cisplatin plus pemetrexed in patients with metastatic lung adenocarcinoma with EGFR mutations. J. Clin. Oncol. 31:3327–3334. 23816960 10.1200/JCO.2012.44.2806

[8] Wu, Y. L. , C. Zhou , C. P. Hu , J. Feng , S. Lu , Y. Huang , et al. 2014 Afatinib versus cisplatin plus gemcitabine for first‐line treatment of Asian patients with advanced non‐small‐cell lung cancer harbouring EGFR mutations (LUX‐Lung 6): an open‐label, randomised phase 3 trial. Lancet Oncol. 15:213–222. 24439929 10.1016/S1470-2045(13)70604-1

[9] Cataldo, V. D. , D. L. Gibbons , R. Perez‐Soler , and A. Quintás‐Cardama 2011 Treatment of non‐small‐cell lung cancer with erlotinib or gefitinib. N. Engl. J. Med. 364:947–955. 21388312 10.1056/NEJMct0807960

[10] Rosell, R. , T. G. Bivona , and N. Karachaliou . 2013 Genetics and biomarkers in personalisation of lung cancer treatment. Lancet 382:720–731. 23972815 10.1016/S0140-6736(13)61715-8

[11] Yu, H. A. , M. E. Arcila , N. Rekhtman , C. S. Sima , M. F. Zakowski , W. Pao , et al. 2013 Analysis of tumor specimens at the time of acquired resistance to EGFR‐TKI therapy in 155 patients with EGFR‐mutant lung cancers. Clin. Cancer Res. 19:2240–2247. 23470965 10.1158/1078-0432.CCR-12-2246 PMC3630270

[12] Aparicio, S. , and C. Caldas . 2013 The implications of clonal genome evolution for cancer medicine. N. Engl. J. Med. 368:842–851. 23445095 10.1056/NEJMra1204892

[13] Gerlinger, M. , A. J. Rowan , S. Horswell , J. Larkin , D. Endesfelder , E. Gronroos , et al. 2012 Intratumor heterogeneity and branched evolution revealed by multiregion sequencing. N. Engl. J. Med. 366:883–892. 22397650 10.1056/NEJMoa1113205 PMC4878653

[14] de Bruin, E. C. , N. McGranahan , R. Mitter , M. Salm , D. C. Wedge , L. Yates , et al. 2014 Spatial and temporal diversity in genomic instability processes defines lung cancer evolution. Science 346:251–256. 25301630 10.1126/science.1253462 PMC4636050

[15] Zhang, J. , J. Fujimoto , J. Zhang , D. C. Wedge , X. Song , J. Zhang , et al. 2014 Intratumor heterogeneity in localized lung adenocarcinomas delineated by multiregion sequencing. Science 346:256–259. 25301631 10.1126/science.1256930 PMC4354858

[16] Leary, R. J. , M. Sausen , I. Kinde , N. Papadopoulos , J. D. Carpten , D. Craig , et al. 2012 Detection of chromosomal alterations in the circulation of cancer patients with whole‐genome sequencing. Sci. Transl. Med. 4:154–162. 10.1126/scitranslmed.3004742 PMC3641759 23197571

[17] Chan, K. C. , P. Jiang , Y. W. Zheng , G. J. Liao , H. Sun , J. Wong , et al. 2013 Cancer genome scanning in plasma: detection of tumor‐associated copy number aberrations, single‐nucleotide variants, and tumoral heterogeneity by massively parallel sequencing. Clin. Chem. 59:211–224. 23065472 10.1373/clinchem.2012.196014

[18] Punnoose, E. A. , S. Atwal , W. Liu , R. Raja , B. M. Fine , B. G. Hughes , et al. 2012 Evaluation of circulating tumor cells and circulating tumor DNA in non‐small cell lung cancer: association with clinical endpoints in a phase ii clinical trial of pertuzumab and erlotinib. Clin. Cancer Res. 18:2391–2401. 22492982 10.1158/1078-0432.CCR-11-3148

[19] Luo, J. , L. Shen , and D. Zheng . 2014 Diagnostic value of circulating free DNA for the detection of EGFR mutation status in NSCLC: a systematic review and meta‐analysis. Sci. Rep. 4:6269. 25201768 10.1038/srep06269 PMC5385820

[20] Kuang, Y. , A. Rogers , B. Y. Yeap , L. Wang , M. Makrigiorgos , K. Vetrand , et al. 2009 Noninvasive detection of EGFR T790m in gefitinib or erlotinib resistant non‐small cell lung cancer. Clin. Cancer Res. 15:2630–2636. 19351754 10.1158/1078-0432.CCR-08-2592 PMC2727796

[21] Sakai, K. , A. Horiike , D. L. Irwin , K. Kudo , Y. Fujita , A. Tanimoto , et al. 2013 Detection of epidermal growth factor receptor T790M mutation in plasma DNA from patients refractory to epidermal growth factor receptor tyrosine kinase inhibitor. Cancer Sci. 104:1198–1204. 23721103 10.1111/cas.12211 PMC7656545

[22] Maheswaran, S. , L. V. Sequist , S. Nagrath , L. Ulkus , B. Brannigan , C. V. Collura , et al. 2008 Detection of mutations in EGFR in circulating lung‐cancer cells. N. Engl. J. Med. 359:366–377. 18596266 10.1056/NEJMoa0800668 PMC3551471

[23] Sorensen, B. S. , L. Wu , W. Wei , J. Tsai , B. Weber , E. Nexo , et al. 2014 Monitoring of epidermal growth factor receptor tyrosine kinase inhibitor‐sensitizing and resistance mutations in the plasma DNA of patients with advanced non‐small cell lung cancer during treatment with erlotinib. Cancer 120:3896–3901. 25103305 10.1002/cncr.28964 PMC4303984

[24] Jackman, D. , W. Pao , G. J. Riely , J. A. Engelman , M. G. Kris , P. A. Jänne , et al. 2010 Clinical definition of acquired resistance to epidermal growth factor receptor tyrosine kinase inhibitors in non‐small‐cell lung cancer. J. Clin. Oncol. 28:357–360. 19949011 10.1200/JCO.2009.24.7049 PMC3870288

[25] Yang, J. J. , H. J. Chen , H. H. Yan , X. C. Zhang , Q. Zhou , J. Su , et al. 2013 Clinical modes of EGFR tyrosine kinase inhibitor failure and subsequent management in advanced non‐small cell lung cancer. Lung Cancer 79:33–39. 23079155 10.1016/j.lungcan.2012.09.016

[26] Kobayashi, S. , T. J. Boggon , T. Dayaram , P. A. Jänne , O. Kocher , M. Meyerson , et al. 2005 EGFR mutation and resistance of non–small‐cell lung cancer to gefitinib. N. Engl. J. Med. 352:786–792. 15728811 10.1056/NEJMoa044238

[27] Balak, M. N. , Y. Gong , G. J. Riely , R. Somwar , A. R. Li , M. F. Zakowski , et al. 2006 Novel D761Y and common secondary T790M mutations in epidermal growth factor receptor‐mutant lung adenocarcinomas with acquired resistance to kinase inhibitors. Clin. Cancer Res. 12:6494–6501. 17085664 10.1158/1078-0432.CCR-06-1570

[28] Oxnard, G. R. , M. F. Paweletz , Y. Kuang , S. L. Mach , A. O'Connell , M. M. Messineo , et al. 2014 Noninvasive detection of response and resistance in EGFR‐mutant lung cancer using quantitative next‐generation genotyping of cell‐free plasma DNA. Clin. Cancer Res. 20:1698–1705. 24429876 10.1158/1078-0432.CCR-13-2482 PMC3959249

[29] Qin, L. , W. Zhong , L. Zhang , L. Y. Li , and M. Z. Wang , 2011 Comparison of three methods for detecting epidermal growth factor receptor mutations in plasma DNA samples of Chinese patients with advanced non‐small cell lung cancer. Chin. Med. J. (Engl). 124:887–891. 21518597

[30] Oxnard, G. R. , M. E. Arcila , C. S. Sima , G. J. Riely , J. Chmielecki , M. G. Kris , et al. 2011 Acquired resistance to EGFR tyrosine kinase inhibitors in EGFR‐mutant lung cancer: distinct natural history of patients with tumors harboring the T790M mutation. Clin. Cancer Res. 17:1616–1622. 21135146 10.1158/1078-0432.CCR-10-2692 PMC3060283

[31] Hata, A. , N. Katakami , H. Yoshioka , J. Takeshita , K. Tanaka , S. Nanjo , et al. 2013 Rebiopsy of non‐small cell lung cancer patients with acquired resistance to epidermal growth factor receptor‐tyrosine kinase inhibitor. Cancer 119:4325–4332. 24105277 10.1002/cncr.28364

[32] Li, W. , S. Ren , J. Li , A. Li , L. Fan , X. Li , et al. 2014 T790M mutation is associated with better efficacy of treatment beyond progression with EGFR‐TKI in advanced NSCLC patients. Lung Cancer 84:295–300. 24685306 10.1016/j.lungcan.2014.03.011

[33] Zheng, D. , X. Ye , M. Z. Zhang , Y. Sun , J. Y. Wang , J. Ni , et al. 2016 Plasma EGFR T790M ctDNA status is associated with clinical outcome in advanced NSCLC patients with acquired EGFR‐TKI resistance. Sci. Rep. 12:20913. 10.1038/srep20913 PMC4751431 26867973

[34] Mok, T. S. , Y. L. Wu , J. S. Lee , C. J. Yu , V. Sriuranpong , et al. 2015 Detection and dynamic changes of EGFR mutations from circulating tumor DNA as a predictor of survival outcomes in NSCLC patients treated with first‐line intercalated erlotinib and chemotherapy. Clin. Cancer Res. 21:3196–3203. 25829397 10.1158/1078-0432.CCR-14-2594

[35] Wang, Z. , R. Chen , S. Wang , J. Zhong , M. Wu , J. Zhao , et al. 2015 Quantification and dynamic monitoring of EGFR T790M in plasma cell‐free DNA by digital PCR for prognosis of EGFR‐TKI treatment in advanced NSCLC. PLoS ONE 9:e110780. 10.1371/journal.pone.0110780 PMC4236040 25405807

[36] Ma, M. , C. Shi , J. Qian , J. Teng , H. Zhong , and B. Han . 2016 Comparison of plasma and tissue samples in epidermal growth factor receptor mutation by ARMS in advanced non‐small cell lung cancer. Gene 591:58–64. 27370697 10.1016/j.gene.2016.06.053

[37] Hindson, C. M. , J. R. Chevillet , H. A. Briggs , E. N. Gallichotte , I. K. Ruf , B. J. Hindson , et al. 2013 Absolute quantification by droplet digital PCR versus analog real‐time PCR. Nat. Methods 10:1003–1005. 23995387 10.1038/nmeth.2633 PMC4118677

[38] Thress, K. S. , R. Brant , T. H. Carr , S. Dearden , S. Jenkins , H. Brown , et al. 2014 EGFR mutation detection in ctDNA from NSCLC patient plasma: a cross‐platform comparison of technologies to support the clinical development of AZD9291. J. Clin. Oncol. 9:509–515 10.1016/j.lungcan.2015.10.004 26494259

[39] Takahama, T. , K. Sakai , M. Takeda , K. Azuma , T. Hida , M. Hirabayashi , et al. 2016 Detection of the T790M mutation of EGFR in plasma of advanced non‐small cell lung cancer patients with acquired resistance to tyrosine kinase inhibitors (West Japan oncology group 8014LTR study). Oncotarget 7:58492–58499. doi: 10.18632/oncotarget.11303. 27542267 10.18632/oncotarget.11303 PMC5295446

[40] Lee, Y. , G. K. Lee , Y. S. Lee , W. Zhang , J. A. Hwang , B. H. Nam , et al. 2014 Clinical outcome according to the level of preexisting epidermal growth factor receptor T790M mutation in patients with lung cancer harboring sensitive epidermal growth factor receptor mutations. Cancer 120:2090–2098. 24737599 10.1002/cncr.28711

[41] Park, K. , C. M. Tsai , M. J. Ahn , C. J. Yu , S. W. Kim , V. Sriuranpong , et al. 2012 ASPIRATION: phase II study of continued erlotinib beyond RECIST progression in Asian patients (pts) with epidermal growth factor receptor (EGFR) mutation‐positive non‐small cell lung cancer (NSCLC). J. Clin. Oncol. 30(Suppl. 15) [Abstr TPS7614], http://meetinglibrary.asco.org/content/96347-114.

[42] Soria, J. C. , Y. L. Wu , K. Nakagawa , S. W. Kim , J. J. Yang , M. J. Ahn , et al. 2015 Gefitinib plus chemotherapy versus placebo plus chemotherapy in EGFR‐mutation‐positive non‐small‐cell lung cancer after progression on first‐line gefitinib (IMPRESS): a phase 3 randomised trial. Lancet Oncol. 16:990–998. 26159065 10.1016/S1470-2045(15)00121-7

[43] Greig, S. L. 2016 Osimertinib: First Global Approval. Drugs 76:263–273. 26729184 10.1007/s40265-015-0533-4

